# Alpha-synuclein activates the classical complement pathway and mediates complement-dependent cell toxicity

**DOI:** 10.1186/s12974-021-02225-9

**Published:** 2021-08-16

**Authors:** Emil Gregersen, Cristine Betzer, Woojin S. Kim, Gergo Kovacs, Lasse Reimer, Glenda M. Halliday, Steffen Thiel, Poul Henning Jensen

**Affiliations:** 1 DANDRITE, The Danish Research Institute of Translational Neuroscience, Aarhus University, Aarhus C, Denmark; 2 Department of Biomedicine, Aarhus University, Aarhus C, Denmark; 3 Brain and Mind Centre & Faculty of Medicine and Health, School of Medical Sciences, The University of Sydney, Camperdown, New South Wales Australia

**Keywords:** α-Synuclein, Complement system, MSA, Neurodegeneration, SH-SY5Y, RaCI, Cp20

## Abstract

**Background:**

Synucleinopathies are characterized by neurodegeneration and deposition of the presynaptic protein α-synuclein in pathological protein inclusions. Growing evidence suggests the complement system not only has physiological functions in the central nervous system, but also is involved in mediating the pathological loss of synapses in Alzheimer’s disease. However, it is not established whether the complement system has a similar role in the diseases Parkinson's disease, Dementia with Lewy bodies, and multiple system atrophy (MSA) that are associated with α-synuclein aggregate pathology.

**Methods:**

To investigate if the complement system has a pathological role in synucleinopathies, we assessed the effect of the complement system on the viability of an α-synuclein expressing cell model and examined direct activation of the complement system by α-synuclein in a plate-based activation assay. Finally, we investigated the levels of the initiator of the classical pathway, C1q, in postmortem brain samples from MSA patients.

**Results:**

We demonstrate that α-synuclein activates the classical complement pathway and mediates complement-dependent toxicity in α-synuclein expressing SH-SY5Y cells. The α-synuclein-dependent cellular toxicity was rescued by the complement inhibitors RaCI (inhibiting C5) and Cp20 (inhibiting C3). Furthermore, we observed a trend for higher levels of C1q in the putamen of MSA subjects than that of controls.

**Conclusion:**

α-Synuclein can activate the classical complement pathway, and the complement system is involved in α-synuclein-dependent cellular cytotoxicity suggesting the system could play a prodegenerative role in synucleinopathies.

**Supplementary Information:**

The online version contains supplementary material available at 10.1186/s12974-021-02225-9.

## Background

The neuronal protein α-synuclein (α-syn) is predominantly located in the pre-synapse, where it is involved in the synaptic vesicle release cycle [1, 2]. α-Syn is genetically and pathologically associated with a group of neurodegenerative diseases, synucleinopathies, which is dominated by Parkinson’s disease (PD), multiple system atrophy (MSA), and dementia with Lewy bodies (DLB) [3, 4]. A common feature for the diseases is the presence of intracellular protein inclusions rich in aggregated α-syn [5]. It is believed that aggregated species of α-syn facilitate the progressive neuropathology by affecting the functionality of the neuron accumulating the aggregates and transmitting the seeding competent α-syn to connected neurons where they in a prion-like manner templated the aggregation of the endogenous α-syn of the recipient neurons [6–8]. Besides α-syn pathology, neurodegeneration in synucleinopathies is accompanied by extensive neuroinflammation, and several studies have linked both the innate and the adaptive immune systems to PD development [9, 10]. This is supported by epidemiological findings that anti-inflammatory treatment, e.g., by ibuprofen, reduces the risk of developing PD [11, 12], supporting a role for immune and associated signaling systems in synucleinopathies [13].

Traditionally, the immune system has been divided into an adaptive and an innate immune system although it now is evident the immune system is very interactive. Part of the innate immune system is represented by the complement system, which is critical for fast recognition and clearance of pathogens, dying cells, and misfolded proteins [14]. Upon recognition of foreign or altered structures in the body, activation of the complement system initiates a number of anti-microbial defense mechanisms: (1) potent inflammatory mediators are released (such as the fragment C5a), (2) the formation of a membrane attack complex (MAC) leading to the formation of a cytotoxic pore in the target cell membrane, and (3) deposited complement fragments C4b and C3b functioning as molecular tags (opsonins) that can interact with complement receptors on immune cells, thereby facilitating engulfment by phagocytes and activation of B-cells. The launch of the complement system occurs when one or more of three convergent pathways of activation are initiated: the classical, the alternative, or the lectin pathway. The classical pathway is initiated by direct binding of C1 to a pathogen or through the binding to IgGs covering a pathogen. C1 is then able to cleave and activate the complement factors C2 and C4, which covalently attach to nearby surfaces via exposed reactive thioesters. This leads to the formation of more active enzyme complexes resulting in the effector functions mentioned above. The C1 complex consists of the recognition molecules C1q and the two proteases C1r and C1s, which are formed by Ca^2+^-dependent interactions [15].

Although the classic view of the complement system is to be active in blood and other body fluids, it is shown to have functions in the central nervous system (CNS) as well. In the developing brain, complement factor C1q facilitates the crucial and physiological clearance of cells and the elimination of synapses [16]. This system can later in life be dysfunctionally activated and be involved in the pathological loss of synapses in Alzheimer’s disease [17]. Recent animal experiments suggest a similar role in synucleinopathies [18]. Lewy bodies in the substantia nigra are immunohistochemically positive for complement factors C3d and C4d, whereas the levels of distinct complement factor isotypes are decreased in cerebrospinal fluid of PD patients [19, 20]. Similarly, C3 levels are decreased in cerebrospinal fluid of MSA subjects [21]. Complement factor C4 and C5 have been identified as binding partners of oligomeric α-syn, and a recent proteome study demonstrated that the complement system is differently expressed in two mouse models of PD [18, 22]. This corroborates a hypothesis where the complement system is involved in α-syn-dependent cell loss in synucleinopathies.

In this study, we demonstrate a direct C1q-dependent activation of the classical complement cascade by α-syn that may be responsible for the complement-dependent activity in human plasma that mediates its cytotoxicity toward α-syn expressing cells. This association between α-syn, a key player in common neurodegenerative diseases, and the innate immune system may form the basis for novel strategies for treating PD and DLB.

## Materials and methods

### Serum and recombinant proteins

All human sera used in this report were drawn from healthy donors (five men (aged 25–60) and three women (aged 28–55)) except for the C1q-depleted serum (Quidel—A509). After clotting of the blood at room temperature, the tubes were centrifuged at 2000 rpm for 10 min at 4°C. The serum was isolated and stored at −80°C. The storage time varied from months to years. Heat inactivation of complement factors was achieved by incubation of a sample in a 56°C water bath for 30 min. Such heat treatment is known to inactivate, e.g., complement factors C2 and Factor B [23]. We did not observe any significant difference in complement activity between donors, which correlates with published literature, where sex and age were found to have minimal effect on the classical complement pathway [24].

Recombinant human α-syn, C-terminally truncated α-syn1-95, and β-synuclein (β-syn) were purified from *E. coli* as described in Lindersson et al., which includes a reverse-phase chromatography step that limits lipid contamination [25]. Full-length Tau expressed in *E. coli* were purified as described by Jensen et al. [26]*.* Size-exclusion chromatography analysis of α-syn was carried out using a Superdex 200 Increase 3.2/300 column (Cytiva—28990946). The size of eluted α-syn was estimated using standard protein weight markers (Sigma Aldrich—MWGF1000). Lipopolysaccharide (LPS) was depleted by running the sample through a Detoxi-gel endotoxin removing column (Thermo Fisher—20344). C1q was purified as described in detail by Tenner et al. [27]. In short, C1q was isolated from human serum by binding to BioRex-70 beads followed by elution with a high-salt gradient.

### Cell experiments

SH-SY5Y cell clones with inducible expression of β-galactosidase (β-gal) or α-syn were a kind gift from Professor Leonidas Stefanis and Associate Professor Kostas Vekrellis, Academy of Athens, Greece. The Tet-off system consists of a Tet-off vector and a pTRE-2 vector encoding α-syn or β-gal [28]. Cells were cultured in RPMI 1640 (Lonza) supplemented with 15% fetal calf serum (FCS, Biowest—S1810), 50 U/mL/50 μg/mL penicillin/streptomycin, 250 μg/mL G418, and 50 μg/mL Hygromycin B and maintained at 37° C, 5% CO_2_. For experiments, SH-SY5Y cells were seeded in poly-l-lysine (Sigma Aldrich—P4707)-coated 96-well plates and differentiated in media containing 15% FCS, 20 μM all-trans retinoic acid (Molecular Probes/Invitrogen), and 1 μg/mL doxycycline (Sigma Aldrich—324385). All-trans retinoic acid was included to make the cells non-mitotic. This allows for longer incubation time and development of α-syn-aggregated pathology, as demonstrated by Betzer et al. [29]. Two days postseeding, media were replaced with RPMI 1640 containing 20 μM all-trans retinoic acid and 15% normal human serum or heat-inactivated human serum. Serum was pooled from at least three donors. In some experiments, 5 μM RaCI (C5 inhibitor, a kind gift from Matthijs Jore, Oxford University) or 20 μM Cp20 (C3 inhibitor, a kind gift from Daniel Ricklin, University of Pennsylvania) was combined with normal human serum media. The simultaneous removal of dox initiated the expression of β-gal or α-syn. On day 12, viability was determined by an MTT assay (Life Technologies).

### Complement activation assay

Complement activation was measured as C4b deposition onto surfaces of microtiter wells using a time-resolved immunofluorometric assay (TRIFMA). 96-well FluoroNunc plates (Thermo Scientific—437958) were coated overnight at 4°C with 100 μL, 5 μg/mL, recombinant α-syn, α-syn(1-95), β-syn, Tau, or carbonic anhydrase (Sigma Aldrich—MWGF1000) before being blocked with human serum albumin (HSA, Statens Serum Institute, Copenhagen, Denmark) at 1 mg/ml in TBS (10 mM Tris, 145 mM NaCl, pH 7.4), for 1 h at room temperature (RT). Wells to be coated with only HSA were kept empty until this step. Following three times washing in TBS with 0.05% TWEEN 20, a serial dilution of normal human serum in 4 mM barbital, 145 mM NaCl, 3.8 mM NaN_3_, pH 7.5 supplemented with either 2 mM CaCl_2_ and 1 mM MgCl_2_ or 10 mM EDTA (*Ca*^*2+*^ *depleted*) was added. The plates were incubated at 37°C for 1 h and subsequently washed three times in 200 μL TBS, 0.05% TWEEN 20, 5 mM Ca^2+^. Complement activation and thus deposition of complement fragments was assessed by adding 1 μg/mL biotinylated rabbit antibody reacting with an epitope exposed on cleaved forms of C4 (granted by Wolbink and colleagues [30]), at 4°C overnight. Following a washing step, the plates were incubated with 0.1 μg/mL Streptavidin-Europium (DELFIA® - 1244-360) in TBS, 0.05% TWEEN 20, 25 μM EDTA for 1 h at RT. After a three final washes in TBS, 0.05% TWEEN 20, 5 mM Ca^2+^, the europium in the wells was quantified by time-resolved fluorometry on a Victor™X3 Plate Reader, after addition of an enhancement buffer: 0.57% (v/v) acetic acid, 1% (w/v) PEG 6000, 0.1% (v/v) Triton X-100, 15 μM β-napthoyltrifluoroacetone, 50 μM tri-n-octylphosphine oxide, pH 3.2.

### Human brain samples and western blotting

Human brain samples were obtained from Sydney Brain Bank and NSW Tissue Resource Centre. Controls were free of neuropathology, while MSA donors were diagnosed clinically and pathologically in accordance with international diagnostic criteria [31]. Brain tissue from the putamen or visual cortex was collected from frozen brain slices using a 3-mm stainless steel biopsy needle. Clinical information (i.e., age, gender, postmortem interval (PMI), disease duration) is summarized in Table 1. For more details on the brain samples, we refer to Don et al. [32]. Tissue was dounce homogenized in ice-cold 250 mM sucrose, 10 mM HEPES, 1 mM EDTA, pH 7.4 containing protease inhibitors (1 μg/mL pepstatin-A, 1 mM benzamidine, 1 μg/mL leupeptin, 1 μg/mL aprotinin; Roche Applied Science). Homogenates were centrifuged 800 g, at 4°C for 10 min and the supernatant was collected as the soluble fraction of the brain extracts. Protein, 40 μg, from the soluble fraction was run on SDS-page (8% acrylamide) and transferred to nitrocellulose membranes. Following overnight blocking with PBS containing 5% non-fat dry milk for 2 h at 4°C, the membranes were incubated with primary antibodies (rabbit anti-C1q, DAKO A136, 1/1000 dilution or mouse anti-β-actin, Abcam ab6276, 1/10,000 dilution) in PBS containing 5% non-fat dry milk overnight at 4°C. Next, membranes were washed three times in PBS containing 0.1% Tween 20 before being subjected to secondary antibody conjugated to horseradish peroxidase (anti-rabbit, Dako P0448 or anti-mouse, Bio-Rad 170-6516, 1/2000 dilution) in PBS containing 5% non-fat dry milk for 2 h. Bands were visualized using enhanced chemiluminescence (ECL, GE Healthcare, Buckinghamshire, UK) and X-ray films. Densitometric quantification was performed using ImageJ.

**Table 1:** Demographic information on MSA and control cases

Brain ID	Age	Gender	PMI (hours)	Disease duration (years)
**MSA**				
MSA1	61	Male	21	4
MSA2	82	Male	8	7
MSA3	74	Male	16	10
MSA4	67	Male	11	8
Mean	71 ± 7.8		14 ± 4.9	7.3 ± 2.2
**Control**				
Con1	69	Male	13.5	N/A
Con2	85	Male	9	N/A
Con3	65	Male	14.5	N/A
Con4	88	Male	9	N/A
Mean	76.8 ± 9.9		11.5 ± 2.5	

*PMI* postmortem interval. There was no statistically significant difference in age and PMI between MSA and controls. Statistical test: Student’s *t*-test

### Statistics

Initially, a Shapiro-Wilk test was used to determine if data fitted a normal distribution. When comparing two groups, a ratio paired Student’s *t*-test was used to test the null hypothesis. For multiple comparisons, one-way ANOVA followed by Tukey’s multiple comparison test was conducted. *P*-values < 0.05 were considered statistically significant. All statistical analyses were performed using GraphPad Prism 8.4.1.

## Results

### Alpha-synuclein activates the classical complement cascade

**Fig. 1:**
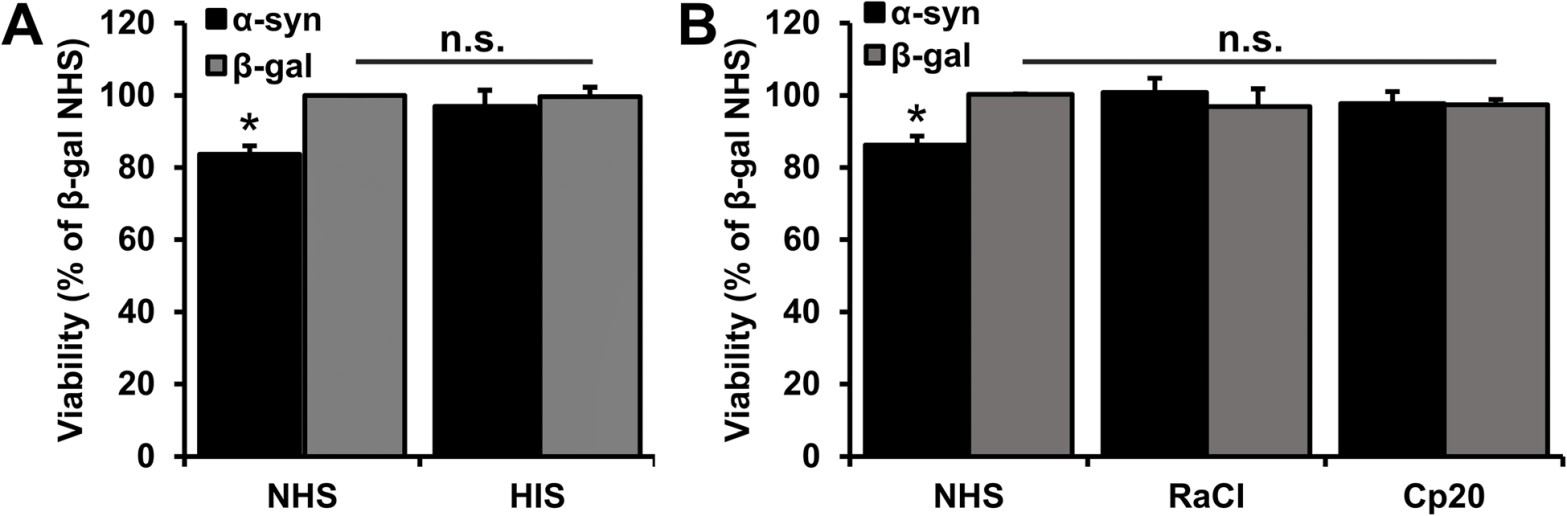
Complement inhibitors rescue alpha-synuclein-induced toxicity in SH-SY5Y cells. **A** Non-mitotic transgenic SH-SY5Y cells expressing α-synuclein (α-syn) or β-galactosidase (β-gal) were cultured in 15% normal human serum (NHS) or heat-inactivated human serum (HIS) for 10 days before viability was measured by an MTT assay. Viability was normalized to the control β-gal expression cells cultured in NHS. **B** Same as in **A** but cells were cultured in NHS without or in the presence of complement cascade inhibitors RaCI or Cp20. The bars represent the mean ± SD of three independent experiments. Statistical test: one-way ANOVA followed by Tukey’s multiple comparison test. n.s. not significant, *P < 0.05

The complement system has been implicated in the neurodegeneration associated with AD. We hypothesized that it also contributes to the neurodegeneration in the synucleinopathies; PD, DLB, and MSA that are characterized by α-syn aggregate associated neurodegeneration and neuroinflammation. To test the hypothesis, we cultivated human SH-SY5Y cells under non-mitotic conditions and induced expression of α-syn by removal of doxycycline to induce α-syn-dependent cell stress [29]. Heat inactivation of serum is used as a routine method to inactivate the complement system in cell culture supplement. Culturing SH-SY5Y cells expressing α-syn for 10 days in the presence of not heat-inactivated human serum resulted in a 15% reduction in viability compared to SH-SY5Y cells expressing β-gal as a negative control protein (Fig. 1A). In contrast, no α-syn-dependent reduction in viability was observed when the cells were cultured in the presence of human serum where the complement system had been inactivated by heat treatment. To corroborate a role for complement-dependent components in α-syn-induced cytotoxicity, we tested the effect of two specific inhibitors of complement components: RaCI for complement component C5 [33] and CP20 for complement component C3 [34]. Addition of either inhibitor to the culture medium containing non-heat-inactivated serum completely rescued the α-syn-dependent cell loss without exerting toxicity on the β-gal expressing cells (Fig. 1B). This demonstrates that cellular α-syn stress triggers a complement-dependent cytotoxic pathway that contributes to the demise of the cells.

**Fig. 2:**
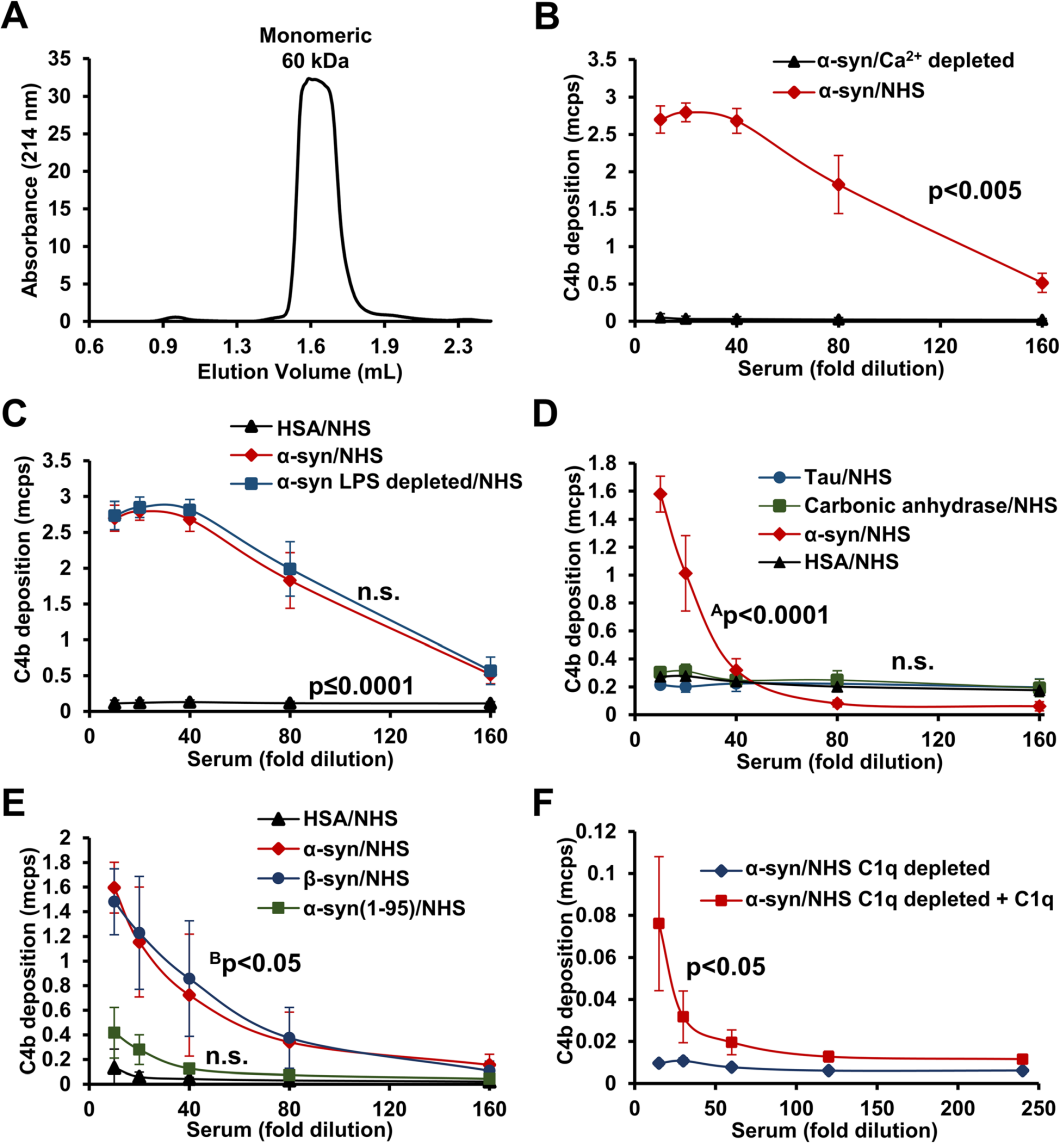
Alpha-synuclein activates the classical complement cascade. **A** Absorbance profile at 214 nm of purified α-synuclein (α-syn) subjected to size-exclusion chromatography analysis. Monomeric α-syn elutes as a 60-kDa protein due to its natively unfolded state. **B** Wells coated with α-syn were incubated with normal human serum (NHS) or NHS in the presence of EDTA (Ca^2+^ depleted). The deposition of complement was measured using a biotinylated C4b-fragment-specific antibody followed by incubation with streptavidin-europium. Time-resolved fluorescence from the europium is given as million counts per second (mcps). Statistical test: area under the curve (AUC) was compared by a ratio paired *t*-test. **C** Same procedure as **B** except wells were coated with either human serum albumin (HSA), α-syn, or α-syn depleted for lipopolysaccharide (LPS depleted) followed by incubation with NHS. Statistical test: AUC was compared by a one-way ANOVA for repeated measures followed by Tukey’s multiple comparison. **D** Same as **B**, except wells were coated with HSA, α-syn, Tau, or carbonic anhydrase followed by incubation with NHS. Statistical test: AUC was compared by a one-way ANOVA for repeated measures followed by Tukey’s multiple comparison. ^A^α-Syn compared to Tau, carbonic anhydrase, and HSA. **E** Same as **B**, except wells were coated with HSA, α-syn, β-synuclein (β-syn), or C-terminal truncated α-syn (α-syn(1-95)) followed by incubation with NHS. Statistical test: AUC was compared by a one-way ANOVA for repeated measures followed by Tukey’s multiple comparison. ^B^α-Syn and β-syn compared to HSA. **F** Same as **B**, except α-syn-coated wells were incubated with C1q-depleted NHS (C1q depleted) without or with added C1q. Statistical test: AUC was compared by a ratio paired *t*-test. All data points are presented as mean ± SD. **B**, **C**, **E**, **F** *n* = 3; **D** *n* = 5

To investigate if α-syn directly can activate the complement system, we used a plate-based immunoassay to investigate if immobilized α-syn can stimulate the complement-dependent deposition of complement factor C4b on the surface of α-syn-coated microtiter plates. Size-exclusion chromatography analysis showed the purified α-syn used for coating was almost exclusively monomeric (>98%) (Fig. 2A). α-Syn stimulates the deposition of C4b when co-incubated with non-heat-inactivated human serum. The activation of complement in serum is calcium dependent, and the deposition of C4 fragments was abolished in Ca^2+^-free buffer (Fig. 2B). The α-syn used for the experiment is expressed in *E. coli*, so contaminating lipopolysaccharide (LPS) could in principle be responsible for the activation, although this is primarily known for the C3b depositing alternative pathway. However, treatment of the α-syn with an LPS removal kit had no effect on the complement activation (Fig. 2C). Carbonic anhydrase and Alzheimer’s disease-associated Tau did not facilitate C4b deposition (Fig. 2D). This emphasizes the specificity of α-syn in activating the complement system. Interestingly, β-synuclein (β-syn) were equally capable of activating the complement system (Fig. 2E). The C-terminal part of α-syn is important in mediating the C4b deposition, as the activation was greatly reduced when coating with a C-terminally truncated α-syn(1-95) (Fig. 2E). To investigate if α-syn-dependent deposition of C4 fragments was mediated by the classical pathway, we tested the effect of immune-depleting C1q, the first initial component of the classical complement pathway from the activation assay. We found C1q was essential for activation by α-syn, as no deposition of C4b was present when using C1q-depleted human sera (Fig. 2F). This deposition could be partially recovered by adding physiological levels of C1q to the serum. Together, our data demonstrate that α-syn can activate the classical complement pathway by acting on early steps in the cascade.

### C1q levels in multiple system atrophy patients

**Fig. 3:**
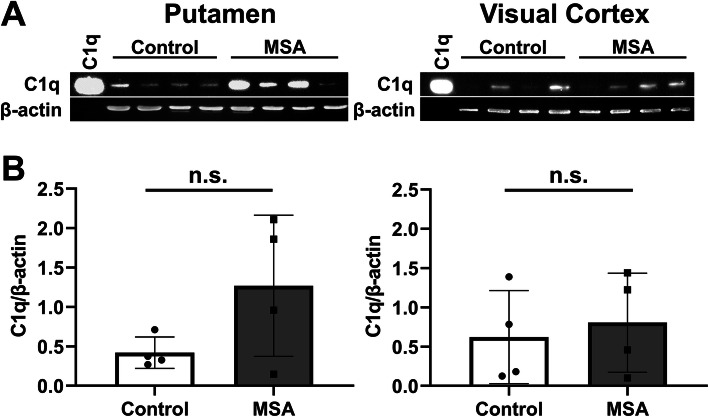
Comparison of complement factor C1q levels in the putamen and visual cortex in healthy controls and multiple system atrophy patients. **A** Tissue from the putamen and visual cortex from 4 patient brains affected by multiple system atrophy (MSA) patients and 4 brains of healthy age-matched controls were extracted, resolved by SDS-page, and analyzed by immunoblotting for the presence of C1q and β-actin. Purified C1q was included as a positive control. **B** C1q and β-actin bands were quantified by densitometry using ImageJ and displayed as the ratio between the two. Points refer to the individual samples, while the column displays mean ± SD. Statistical test: mean compared by Welch’s *t* test. n.s. not significant. Full blots can be found in the supplementary material

MSA is a rare, aggressive neurodegenerative disease that is characterized by the development of α-syn aggregate containing inclusions in oligodendrocytes, neurodegeneration, and a strong neuroinflammation. To assess if activation of the classical complement pathway could be involved in MSA pathogenesis, we evaluated the levels of C1q in the soluble fraction of postmortem tissue extracts from four MSA patients, versus four age-matched healthy controls by western blotting (Fig. 3). We assessed putamen, a brain area typically affected by α-syn aggregates and neurodegeneration in MSA, and the visual cortex, which is generally unaffected. The band visualized on the immunoblot in Fig. 3 represents the A-chain of complement factor C1q. The analysis showed a trend toward higher levels of C1q in the putamen of individuals with MSA, where three MSA patients exhibited high C1q levels compared to neurological healthy individuals, but it did not reach statistical significance (*P* = 0.15). The C1q levels in the visual cortex were similar for MSA and control samples (*P* = 0.68).

In summary, we have shown that α-syn directly activates the classical complement pathway and in cells confers a complement-dependent component to the molecular α-syn cytotoxicity. Moreover, in MSA patients, C1q levels tended to be increased in the putamen that is affected by this disease.

## Discussion

The complement system is a critical component in the development and homeostasis of neuronal networks in the CNS [16, 35] where C1 and C3 may serve as eat-me signals for microglia-dependent nibbling of synapses [35–37]. It may also play a detrimental role in diseased states as the β-amyloid mediated loss of synapses in an Alzheimer’s disease mouse model was dependent on complement activation [17], and increased complement activation has been demonstrated in the substantia nigra of PD patients [38]. Our data support a hypothesis whereby α-syn-dependent complement activation in neurons can contribute to the microglia activation due to increased C3b deposition.

We report as a novel finding that immobilized α-syn directly can activate the classical complement pathway in vitro and demonstrate that cellular expression of α-syn contributes to complement-dependent cytotoxicity in a neuronal cell line. The activation relied on cellular α-syn expression and may depend on its binding to the cell surfaces that hold receptors for the α-syn [39–42].

**Fig. 4:**
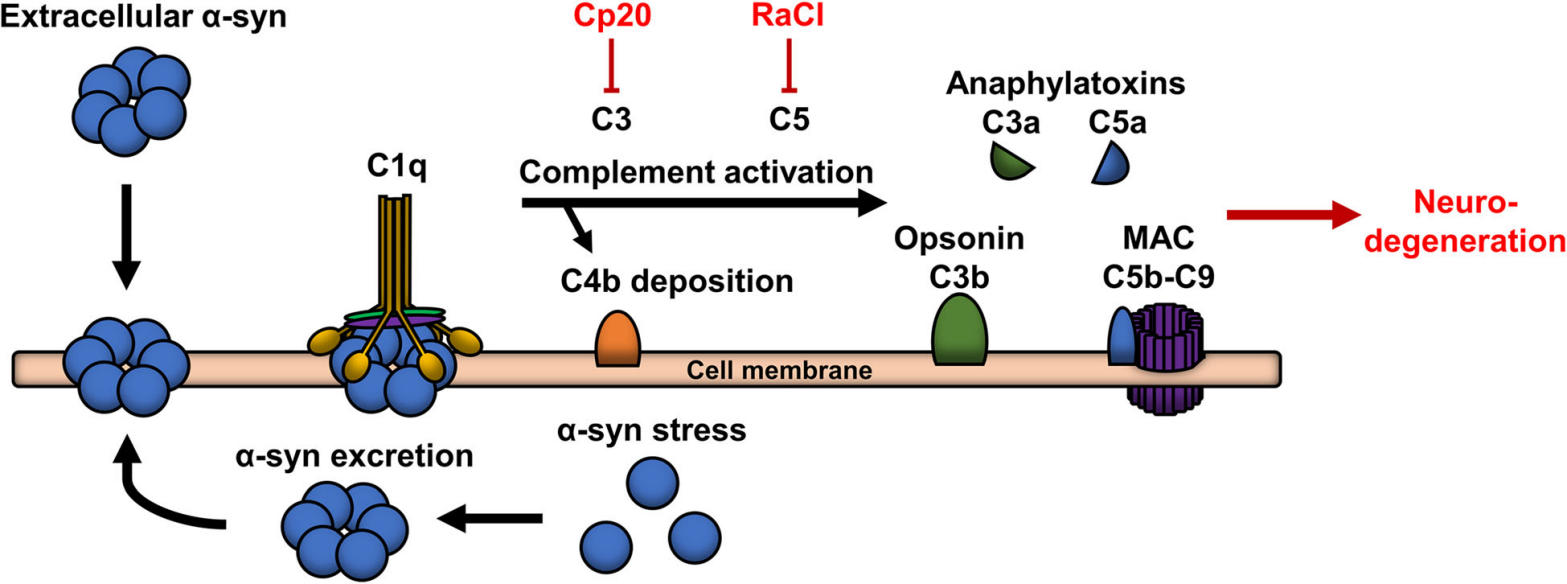
Hypothetical mechanism for α-synuclein mediated complement-dependent cytotoxicity. Intracellular α-synuclein (α-syn) stress, excretion of α-syn-covered extracellular vesicles, or binding of extracellular α-syn to the cell surface leads to the presentation of α-syn at the plasma membrane. Here, α-syn could potentially activate the classical complement cascade by direct binding of C1 leading to deposition of C4b, production of anaphylatoxins, C3a opsonization, and the formation of the membrane attack complex (MAC). Complement inhibitors, exemplified by Cp20 and RACI, holds neuroprotective potential by making the cell inert to the binding of C1q to membrane-associated α-syn

We found that immobilized α-syn was able to activate the classical pathway, which resulted in the deposition of C4b. In a similar manner, α-syn can be presented on the cell surface by binding of secreted α-syn to cell surface receptors, by the formation of membrane spanning pores, or by its presentation on extracellular vesicles released from cells [43–45]. Here, it can potentially be recognized by C1q, and thereby initiate the classical complement pathway. If not adequately controlled, the complement system can induce cell death by the formation of the membrane attack complex [46] (Fig. 4), a mechanism we find most likely to be active in our cell model that is devoid of immune cells. Such a role is corroborated by the recent demonstration of complement system components to be differently expressed in two different mouse models of PD [18]. Complement activation does not seem to be dependent on the pathological aggregation of α-syn, as the a-syn preparation consisted mostly of monomeric α-syn (Fig. 2A). Moreover, the homologue β-syn, which lacks the NAC region essential for fibrilization, also initiated the complement cascade (Fig. 2E).

In this study, we used human serum as the source for complement factors because it contains all humoral components. The concentrations of the complement factors are higher than in CSF because of the integrity of the blood-brain barrier [47]; however, functional complement factors are produced locally in the brain tissue [16, 48–50] where their concentration and activation likely are regulated by the local microenvironment. Utilizing a plate-based immunoassay to quantify the C4b deposition, we showed that α-syn coated in the microtiter wells activated the complement system in vitro. This is in contrast to a previous study that found the α-syn-112 splice variant but not full-length α-syn activated the complement system [51]. The conflicting results could be due to their quantification of the further downstream C5b-9 as opposed to C4b in the present report. Furthermore, we assured our observation was not caused by contaminating LPS in the recombinant α-syn preparation, but such assessment was not reported in the previous study. C1q depletion of NHS completely abolished α-syn complement deposition while the addition of C1q partly restored the signal (Fig. 2C). We assume that the absence of complete recovery of the complement activation was due to a possible difference in concentrations of, e.g., C1, C1s, or C4. Nevertheless, taken together, this data strongly suggest the classical pathway is activated by α-syn.

To support a role for complement activation in synucleinopathies, we compared C1q levels in tissue from the putamen and visual cortex from MSA patients and controls. MSA is characterized by a strong accumulation of α-syn pathology in the putamen but not the visual cortex. We observed increased putaminal C1q levels in three of the four MSA cases. Statistical significance was not achieved due to one case being very low. However, the observation justifies larger comparative studies of complement levels not only in brain tissue affected by MSA but also in other synucleinopathies like PD and DLB.

The realization that the complement system is involved in numerous disease states has led to therapeutic strategies that modulate specific complement components [52]. Our demonstration of targeting C3 and C5 with CP20 and RaCl is protective in our cell model against α-syn-dependent complement-mediated cytotoxicity supports complement targeting disease-modifying strategies also may hold potential in PD and other synucleinopathies.

## Conclusion

In conclusion, our results demonstrate that α-syn can activate the classical pathway of the complement system and this system contributes to α-syn-dependent cytotoxicity. This suggests the complement system contributes to the neurodegeneration of MSA and related synucleinopathies.

## Supplementary Information

**Additional file 1.** Full western blot from Figure 3 with protein ladder.

## Data Availability

All relevant data are available upon request directed to the corresponding author.

## Abbreviations

α-syn: α-Synuclein
β-gal: β-Galactosidase
β-syn: β-Synuclein
CNS: Central nervous system
DLB: Dementia with Lewy bodies
FCS: Fetal calf serum
HIS: Heat-inactivated serum
HSA: Human serum albumin
LPS: Lipopolysaccharide
MAC: Membrane attack complex
MSA: Multiple system atrophy
NHS: Normal human serum
PD: Parkinson’s disease
RT: Room temperature
TRIFMA: Time-resolved immunofluorometric assay
