# Supplementary material for: Alpha-synuclein activates the classical complement pathway and mediates complement-dependent cell toxicity
Source: J Neuroinflammation. 2021 Aug 16;18:177. doi: 10.1186/s12974-021-02225-9 (PMC8369722; doi:10.1186/s12974-021-02225-9)

**Supplementary materials**

Full western blot from Figure 3 with protein ladder.

Putamen - C1q

**Control**

**C1q**

**MSA**





Putamen – β-actin

**Control**

**C1q**

**MSA**


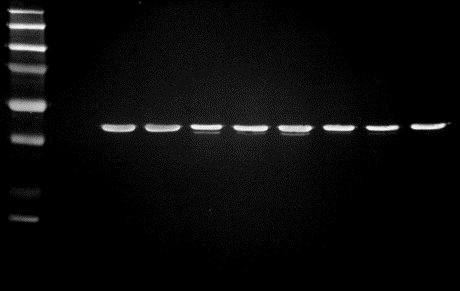


Visual Cortex – C1q

**Control**

**C1q**

**MSA**


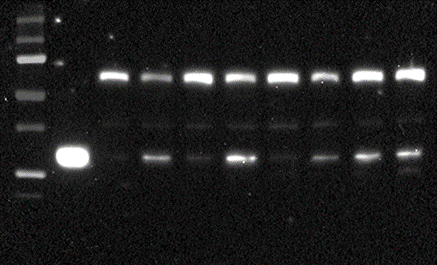


Visual Cortex – β-actin

**Control**

**C1q**

**MSA**


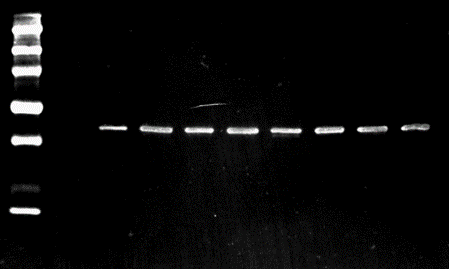

Supplement: Supplementary file 1 — Additional file 1. Full western blot from Figure 3 with protein ladder. [file 12974_2021_2225_MOESM1_ESM.docx]
